# Calcitriol attenuates diethylnitrosamine-induced hepatic fibrosis in rats by reducing oxidative stress and fibrogenic mediators

**DOI:** 10.1371/journal.pone.0347908

**Published:** 2026-05-06

**Authors:** Komsan Arramrak, Namthip Witayavanitkul, Maneerat Chayanupatkul, Natcha Wanpiyarat, Prasong Siriviriyakul, Duangporn Werawatganon

**Affiliations:** 1 Center of Excellence in Alternative and Complementary Medicine for Gastrointestinal and Liver Diseases, Department of Physiology, Faculty of Medicine, Chulalongkorn University, Bangkok, Thailand; 2 Department of Pathology, Faculty of Medicine, Chulalongkorn University, Bangkok, Thailand; Zhejiang University, CHINA

## Abstract

Vitamin D deficiency is associated with poor outcomes and increased mortality in cirrhosis. Calcitriol, the active form of vitamin D_3_, has antioxidant and hepatoprotective properties; however, its effects on diethylnitrosamine (DEN)-induced liver fibrosis remain unclear. This study investigated the effects of calcitriol on oxidative stress, inflammation, and fibrogenesis in DEN-induced liver fibrosis rat model. Male Sprague-Dawley rats (n = 6/group) were assigned to four groups: control (CON), DEN, DEN + low-dose calcitriol (DEN + LVD_3_: 5 µg/kg BW), and DEN + high-dose calcitriol (DEN + HVD_3_: 10 µg/kg BW). Liver fibrosis was induced by weekly DEN injections (70 mg/kg, i.p.) for 8 weeks. Calcitriol was administered twice weekly (i.p.) throughout the experiment. Oxidative stress (hepatic malondialdehyde; MDA), liver injury (serum ALT, AST), hepatic inflammation (NF-κB p65), antioxidant gene expression (SOD-1, GPX-1), and fibrosis markers (TGF-β1, MMP-12, TIMP-1, α-SMA, collagen) were evaluated by biochemical assays, immunohistochemistry, qRT-PCR, western blotting and H&E and Sirius Red staining. Calcitriol significantly attenuated DEN-induced oxidative stress by decreasing hepatic MDA levels in a dose-dependent manner, and lowered serum ALT and AST levels. It partially enhanced hepatic antioxidant defenses by increasing SOD-1 expression toward control levels and upregulating GPX-1 expression. Calcitriol also markedly suppressed NF-κB p65 activation and fibrotic markers (TGF-β1, MMP-12, α-SMA, collagen), which corresponded with improved histopathological fibrosis scores. TIMP1 expression remained unchanged across all groups. Therefore, calcitriol attenuates DEN-induced liver fibrosis by reducing oxidative stress, suppressing inflammatory and fibrogenic signaling, and enhancing antioxidant defenses.

## Introduction

Liver disease is a major global health concern, causing approximately two million deaths each year from cirrhosis [1], viral hepatitis, and hepatocellular carcinoma (HCC) [2–4]. The Asia-Pacific region, home to more than half of the world’s population, bears a disproportionate burden, accounting for 62.6% of global liver disease-related deaths in 2015, including 54.3% of deaths from cirrhosis, 72.7% from HCC, and over two-thirds of acute viral hepatitis cases [5]. Despite the growing prevalence of chronic liver diseases, no specific antifibrotic therapies are currently available; existing treatments primarily target underlying etiologies and are often insufficient [6]. Increasing evidence indicates that low vitamin D levels are associated with the development and progression of chronic liver diseases [7] including chronic hepatitis B virus (HBV) infection [8], non-alcoholic fatty liver disease (NAFLD) [9], cirrhosis [10], and HCC [11]. Adequate vitamin D status appears to confer protective effects against liver fibrosis in adults with NAFLD [12], whereas vitamin D deficiency is linked to an increased risk of fibrosis progression [13]. Therefore, vitamin D may represent a potential therapeutic strategy for mitigating liver fibrosis.

Calcitriol, or vitamin D_3_ (VD_3_), plays a critical role in regulating genes involved in cell proliferation, differentiation, apoptosis, angiogenesis, and inflammation [14,15]. Its physiological effects are mediated through the vitamin D receptor (VDR), a member of the nuclear hormone receptor family [16] expressed not only in classical target tissues but also in immune cells and non-parenchymal liver cells [17]. Several in *in vivo* studies have demonstrated the hepatoprotective properties of vitamin D_3_. Goto et al. (2022) demonstrated that long-term dietary vitamin D_3_ supplementation (10,000 IU/kg for 25 weeks) significantly reduced the progression of diethylnitrosamine/thioacetamide (DEN/TAA)-induced preneoplastic liver lesions [7]. Importantly, serum calcium and phosphorus levels were not significantly altered, indicating a favorable safety profile [7]. Similarly, Megahed et al. (2023) showed that daily intramuscular vitamin D_3_ (1,000 IU/kg body weight (BW)) for 8 weeks ameliorated TAA-induced hepatotoxicity and fibrosis in rats by reducing oxidative stress, inflammation, and key fibrogenic markers, including transforming growth factor-beta (TGF-β) and alpha-smooth muscle actin (α-SMA) [18]. Ma et al. (2020) further demonstrated that intraperitoneal (i.p.) administration of active vitamin D (1,25[OH]₂D₃) at 5 or 10 μg/kg BW twice weekly for 8 weeks improved high-fat diet-induced NAFLD-related liver fibrosis by reducing oxidative stress and inflammation and inhibited the p53–p21 signaling pathway in rats [19]. In addition, vitamin D_3_ has been shown to enhance hepatic antioxidant defenses by increasing glutathione levels and elevating superoxide dismutase (SOD) and glutathione peroxidase (GPX) activities [20]. Collectively, these findings informed our selection of calcitriol doses (5 and 10 μg/kg BW, administered twice weekly via i.p. injection), which have not previously been evaluated in a DEN-induced liver fibrosis model with an 8-week duration.

DEN also known as N-nitrosodiethylamine, is a well-established hepatotoxin and carcinogen widely used to model the pathological progression of liver fibrosis in experimental animals [21,22]. DEN undergoes metabolic activation to yield reactive intermediates that bind to cellular DNA and proteins, resulting in DNA damage, hepatocellular necrosis, inflammation, neutrophil infiltration, and increased reactive oxygen species (ROS) production [21–23]. These pathological events promote hepatic stellate cell (HSC) activation and excessive extracellular matrix (ECM) deposition, leading to persistent injury and progressive, reproducible fibrosis within a relatively short induction period (typically 4–6 weeks with once-weekly dosing) [24,25]. In addition, DEN administration produces stable fibrosis with low mortality and minimal spontaneous regression, making it a reliable model for studying early-stage human fibrogenesis and for mechanistic or therapeutic investigations [24,26–28]. Chen et al. (2021) reported that intraperitoneal administration of DEN at 70 mg/kg BW once weekly for 9 weeks successfully induced liver fibrosis with low mortality rate [29]. Similarly, DEN at 50 mg/kg BW twice weekly for 7 weeks induced liver cirrhosis [30]. Based on these findings, the present study used DEN at 70 mg/kg BW once weekly for 8 weeks, providing a relatively short induction period while maintaining ethical and economic advantages.

Liver fibrosis is characterized by elevated liver injury markers, including alanine aminotransferase (ALT) and aspartate aminotransferase (AST), increased lipid peroxidation products such as malondialdehyde (MDA), and the upregulation of fibrogenic markers, including TGF-β and α-SMA [18,31,32]. α-SMA reflects activated HSCs and enhanced ECM production, while increased matrix metalloproteinase 12 (MMP-12) expression indicates ongoing ECM remodeling and is often associated with inflammatory sites [33]. Tissue inhibitors of metalloproteinases (TIMPs), particularly TIMP-1, counteract MMP activity and are recognized contributors to fibrosis progression [34]. TIMP-1 levels are consistently elevated in liver tissue and serum in both human liver disease and experimental models, correlating closely with fibrosis severity [35]. SOD, a key antioxidant enzyme that converts superoxide anions (O_2_•^-^) into hydrogen peroxide (H_2_O_2_) and oxygen (O_2_), is reduced in experimental models of liver injury, including methionine- and choline-deficient diet induced NASH in mice [36]. Similarly, DEN-induced fibrosis increases oxidative stress, leading to elevated lipid peroxidation and decreased SOD expression compared with controls [30]. DEN exposure also raises MDA levels, lowers reduced glutathione (GSH) content, and diminishing the activities of GPX [37]. In addition, DEN administration significantly increases histopathological changes and the expression of inflammatory mediators such as IL-1β, TNF-α, and nuclear factor-erythroid 2-related factor-2 (Nrf-2) in rat liver tissue [22]. Taken together, these pathological changes highlight the central role of oxidative stress, inflammation, and fibrogenesis in DEN-induced liver injury. Therefore, this study aimed to investigate the attenuating effects of calcitriol at doses of 5 and 10 μg/kg BW on oxidative stress and fibrosis in a DEN-induced early progressive stage of liver fibrosis in rats. The intervention timing was selected to model a clinically relevant scenario in which treatment is initiated during ongoing disease progression, particularly when etiological therapy is not feasible.

## Materials and methods

### Ethics approval and experimental design

The study was approved by the Institutional Animal Care and Use Committee (IACUC), Faculty of Medicine, Chulalongkorn University, Thailand (Animal ethics approval no. 2491031). Twenty-four male Sprague-Dawley rats (5 weeks old) were obtained from Nomura Siam International Co., Ltd., Thailand. At baseline, all rats were considered healthy based on the absence of clinical signs of illness, normal grooming behavior, and body weight within the expected range for age and strain. No baseline biochemical or histological assessments were performed prior to group allocation. Rats were housed under standard conditions (12-hour light/dark cycle, temperature 25 ± 1 °C) with free access to a standard rodent pellet diet and sterile tap water. After a one-week acclimatization period, animals were randomly assigned to four experimental groups (n = 6/group) as follows: Control group (CON), which received no intervention; Diethylnitrosamine group (DEN); DEN + low-dose calcitriol group (DEN + LVD_3_); and DEN + high-dose calcitriol group (DEN + HVD_3_).

Liver fibrosis was induced via intraperitoneal (i.p.) injections of DEN (70 mg/kg BW: lot. UT2EJ-FL, Tokyo Chemical Industry, Japan) every Tuesday for 8 weeks. Calcitriol was administered exclusively via intraperitoneal (i.p.) injection, twice weekly (Tuesday and Friday) at doses of 5 µg/kg BW (DEN + LVD_3_) or 10 µg/kg BW (DEN + HVD_3_) during the same 8-week period (calcitriol; lot. 2M3190, Cacare®, Nang Kuang Pharmaceutical, Hong Kong, China). All animals were monitored for 5–10 minutes post-injection for signs of distress [38].

According to the Cacare® product information, calcitriol has a half-life (t^1/2^) of 5–8 hours, with pharmacological effects lasting 3–5 days. Vitamin D is expressed International Units (IU), where 1 µg = 40 IU; therefore, the doses of 5 µg and 10 µg correspond to 200 IU and 400 IU, respectively. Previous studies have shown that vitamin D_3_ doses of 2,500−7,000 IU/kg/week in healthy Wistar rats for 4 weeks did not alter biochemical and inflammatory parameters [39], and low-dose vitamin D_3_ (cholecalciferol; 1,500 IU/rat/day for 21 days) produced no histological abnormalities in major organs, such as liver, kidney and spleen [40]. Additionally, Zittermann (2013) reported that an oral intake of up to 750 µg/day for 10 weeks is considered safe in humans [41]. The calcitriol doses (5 and 10 µg/kg BW) were selected based on prior studies demonstrating efficacy and safety in rat models [19]. The 10 µg/kg BW dose was considered the upper effective dose, as higher doses would require intraperitoneal injection volumes exceeding recommended limits and may increase the risk of vitamin D–related toxicity [38].

### Sample size calculation

The sample size for each group was calculated using G*Power software (version 3.1.9.4). The calculation was based on the mean and standard deviation (SD) of hepatic MDA levels reported by Megahed et al., [18], who demonstrated that vitamin D_3_ administration significantly reduce MDA levels in TAA-induced liver fibrosis in rats. Reported MDA levels (mean ± SD) were: control, 60.25 ± 4.38 nmol/g tissue; TAA group, 95.59 ± 21.21 nmol/g tissue; vitamin D_3_ group, 57.50 ± 7.07 nmol/g tissue; and TAA+ vitamin D_3_ group: 77.88 ± 17.11 nmol/g tissue. Using an alpha error of 0.05 and power of 0.95, the analysis indicated that 6 rats/group were required, yielding a total of 24 rats for the study. The detailed G*Power calculation is provided in the Supporting information (S1 Fig).

### Animal welfare and sample collection

Animal health and welfare were monitored twice daily. Humane endpoints criteria triggering euthanasia included signs of infection, chronic inflammation, impaired mobility, abnormal posture, red periorbital staining, respiratory distress, unresponsiveness, failure to groom, or body weight loss exceeding 20% [42]. Animals meeting any of these criteria were humanely euthanized by i.p. sodium thiopental (>50 mg/kg). Deep anesthesia was confirmed by the absence of pedal withdrawal and corneal reflexes prior to euthanasia. No unexpected deaths occurred during the study. Body weight was recorded weekly, and group data are provided in the Supporting information (S2 Fig).

At the end of the 8-week experiment, rats were euthanized using the same procedure. Blood samples were collected by cardiac puncture, and serum was separated by centrifugation at 3,000 × g for 15 minutes at 4 °C. Liver tissues were weighed, then either fixed in 10% formalin for histology (hematoxylin and eosin (H&E), Sirius Red staining), or snap-frozen in liquid nitrogen and stored at −80 °C for subsequent biochemical and molecular analyses.

### Histopathology assessment of liver fibrosis severity

Histopathological evaluation of liver fibrosis was performed using H&E and Sirius Red staining. Liver specimens were randomly selected, fixed in 10% formalin, processed, embedded and sectioned into slices of 5 µm thickness. Sections were deparaffinized in xylene, rehydrated through graded alcohol concentrations, and stained with hematoxylin, followed by differentiation in acid alcohol, rinsing, and eosin counterstaining. Slides were allowed to air-dry overnight before histopathological evaluation [43]. A qualified pathologist, blinded to the experimental groups, meticulously examined all fields in each liver tissue section. Liver fibrosis was evaluated using an adapted METAVIR scoring system (F0–F4) [44,45], which has been commonly applied in rat models of chemically induced liver fibrosis. Although originally developed for human liver disease, the adapted METAVIR scoring system serves as a semi-quantitative tool for assessing relative fibrosis severity and architectural progression in experimental studies. In the present study, METAVIR staging was used for comparative analysis between groups rather than for direct extrapolation to human clinical fibrosis stages.

Sirius Red staining was conducted to evaluate collagen deposition. In stained sections, collagen fibers appeared red, whereas the cytoplasm and nuclei showed a yellow hue. Liver specimens were randomly selected, and 3.5 μm-thick sections were processed and stained with Sirius Red working solution (0.1% Direct Red 80 in saturated picric acid; Sigma, USA). Five random fields per section were captured at 4x magnification under a light microscope [46]. Image analysis was performed using Image J version 1.54f (Fiji Software). Images were converted to an RGB stack, and the green channel was selected (Adjust > RGB stack) to enhance collagen fiber visualization [47]. A threshold was applied to isolate collagen fibers from the background, and the percentage area of collagen deposition was quantified.

### Serum biochemical analysis of ALT and AST

Serum ALT and AST levels were measured using a Dri-Chem NX-600 biochemical analyzer (Fujifilm, Japan) based on spectrophotometric detection. Serum samples were applied to the reagent slide, and results were expressed in units per liter (U/L).

### Measurement of hepatic MDA levels

Hepatic malondialdehyde (MDA), an index of lipid peroxidation, was quantified using a thiobarbituric acid reactive substances (TBARS) assay kit, (Cayman Chemical, USA). Liver tissues were homogenized in RIPA buffer (Cayman Chemical, USA) and centrifuged to obtain supernatants for analysis. Total protein concentration was determined using the Bradford assay (BioRad, USA) at 595 nm. (Skanlt software version3.2, Thermo Fisher Scientific, USA) and expressed as µg/µL. For the TBAR assay, MDA levels were calculated from the TBARS standard curve, with absorbance measured at 532 nm. The hepatic MDA concentrations (µM) were normalized to total protein, and results were expressed as µmol/g protein.

### Western blot analysis

The hepatic expression of MMP-12, TIMP-1, α-SMA, and TGF-β1 were evaluated as markers of ECM deposition and fibrosis. Frozen liver tissues were homogenized in RIPA buffer (Cell Signaling Technology, USA) containing protease and phosphatase inhibitor cocktails (Sigma, USA), as provided in the instructions [48]. Cell membranes were disrupted by sonication (15 seconds on, 10 seconds off, repeated for a total of 65 seconds). Lysates were then centrifuged at 16,000 × g for 5 minutes at 4 ºC, and supernatants were collected for protein quantification using the Bradford assay (BioRad, USA), at 595 nm. (Thermo Fisher Scientific, USA). Equal amounts of protein (30 μg) were separated on 10% SDS-PAGE gels and transferred to PVDF membranes (0.45 μm, BioRad, USA) using a semi-dry transfer system. Membranes were blocked with 1% BSA (Merck, USA) for one hour at room temperature and incubated overnight at 4 °C with primary antibodies against MMP-12 (1:1,000; Novus Biologicals, USA), TIMP-1 (1:1,000; Abcam, USA), α-SMA (1:1,000; Cell Signaling Technology, USA), TGF-β1 (1:1000; Abcam, USA), and cyclophilin B (CPB, 1:20,000, Abcam, USA). After six washes with phosphate buffer saline with tween20 (PBST), membranes were incubated with HRP-conjugated anti-rabbit IgG (1:10,000, Cell Signaling Technology, USA) for 90 minutes at room temperature. Protein bands were visualized using ECL reagents (BioRad, USA) and imaged with the Bio-Rad ChemiDoc Touch Imaging System. Densitometric analysis was performed using Image Lab software (BioRad, USA) and target protein levels were normalized to CPB.

### Tissue microarrays and nuclear factor-κB (NF-κB) p65 immunohistochemistry

Tissue microarray (TMA) technology was used to allow simultaneous analysis of multiple liver tissue samples within a single paraffin block [49]. TMAs were constructed using a Quick-Ray Automated Tissue Microarrayer (Diagnostic Technology, Australia). Recipient blocks were precisely punctured in a specific array pattern, and areas of interest, identified by an experienced pathologist on H&E-stained slides, were matched on the donor blocks, micro-dissected with a 0.6 mm hollow needle, and transferred to recipient block. The completed TMA blocks were sectioned and processed for immunohistochemistry.

For NF-kB p65 immunostaining, liver sections were deparaffinized, rehydrated, and subjected to antigen retrieval using a PT Link system (Dako, USA). Endogenous peroxidase activity was blocked with 3% hydrogen peroxide (H_2_O_2_) for 20 minutes. The section slides were incubated with NF-κB p65 primary antibody (1:400, Cell Signaling Technology, USA) for 60 minutes at room temperature, followed by the Dako REAL EnVision Detection System, Peroxidase/DAB + , HRP Rabbit/Mouse (Dako, USA) for 30 minutes. Visualization was achieved with Dako REAL DAB+ chromogen and substrate buffer (Dako, USA), producing a brown reaction product, and slides were counterstained with hematoxylin. Digital images were captured using a light microscope and analyzed with Aperio Image Scope software (Leica Biosystems Imaging, USA). For quantification, ten random high-power fields per sample were assessed, and NF-kB p65 expression was expressed as the ratio of positive pixels to total pixels [50].

### Total RNA extraction, cDNA synthesis, and Quantitative RT-PCR analysis

Total RNA was extracted from liver tissues using the PureLink™ RNA Mini Kit combined with TRIzol reagent (Invitrogen, USA), followed by DNase I treatment (Thermo Scientific, USA) to eliminate genomic DNA contamination [51]. First-strand cDNA was synthesized from 1 µg of total RNA using the iScript Reverse Transcription Supermix (Bio-Rad, USA) under the following conditions: 25 °C for 5 min (priming), 46 °C for 20 min (reverse transcription), and 95 °C for 1 min (enzyme inactivation). Quantitative RT-PCR was performed using SsoAdvanced Universal SYBR Green Supermix (Bio-Rad, USA) on a QuantStudio 6 Flex Real-Time PCR System (Applied Biosystems, USA). The thermal cycling protocols included an initial denaturation at 95 °C for 5 min, followed by 40 cycles of 95 °C for 15 s and 60 °C for 1 min. Beta actin (β-actin) was used as internal control. Primer sequences for target genes are provided in the Supporting information (S1 Table). Relative gene expression levels were calculated using the 2^−ΔΔCT^ method.

### Statistical analysis

Continuous variables were analyzed using one-way analysis of variance (ANOVA) followed by Turkey’s post hoc test. Categorical data (fibrosis scores) were compared using the Chi-square test. Data are presented as mean ± standard deviation (SD), and statistical significance was defined as *p* < 0.05. All analyses were performed using SPSS software (version 29, USA).

## Results

### Effects of calcitriol on body weight, liver weight, and liver index in DEN-induced liver fibrosis

**Fig 1:**
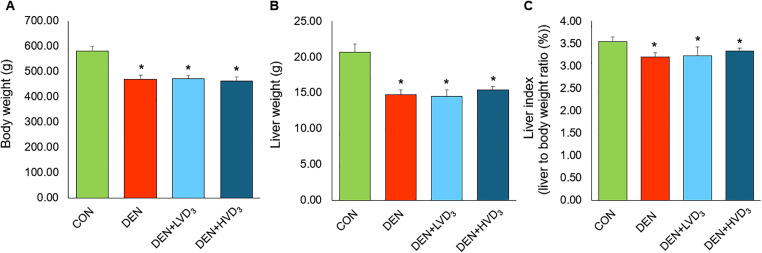
Effects of calcitriol on body weight, liver weight, and liver index in DEN-induced liver fibrosis. **(A)** Body weight, **(B)** liver weight, and **(C)** liver index in the CON, DEN, DEN + LVD₃, and DEN + HVD₃ groups after 8 weeks. All DEN-treated groups showed significantly lower body weight, liver weight, and liver index compared with CON, with no differences among DEN, DEN + LVD₃, and DEN + HVD₃. Data are presented as mean ± SD, n = 6/group. *p < 0.05 compared with the CON group.

After 8 weeks, all DEN-treated groups, including those receiving low- and high-dose calcitriol, exhibited significantly decreased body weight compared with the CON group (DEN: 469.00 ± 15.90 g vs. DEN + LVD_3_: 471.83 ± 11.46 g vs. DEN + HVD_3_: 462.50 ± 15.50 g vs. CON: 582.00 ± 17.23 g, respectively; *p* < 0.05) as shown in Fig 1A.

A similar pattern was observed for liver weight and liver index (liver weight/body weight), with all DEN-treated groups showing significant reductions compared with the CON group (Fig 1B; liver weight; DEN: 14.80 ± 0.65 g, DEN + LVD_3_: 14.53 ± 0.90 g, DEN + HVD_3_: 15.39 ± 0.52 g vs. CON: 20.66 ± 1.19 g, respectively; *p* < 0.05) and liver index; DEN: 3.21 ± 0.09%, DEN + LVD_3_: 3.23 ± 0.20%, DEN + HVD_3_: 3.34 ± 0.06% vs. CON: 3.54 ± 0.10%, respectively; *p* < 0.05 as shown in Fig 1C). However, no significant differences were observed among the DEN, DEN + LVD₃, and DEN + HVD₃ groups for any of these parameters.

### Effects of calcitriol on serum ALT, AST and hepatic MDA levels in DEN-induced liver fibrosis

**Fig 2:**
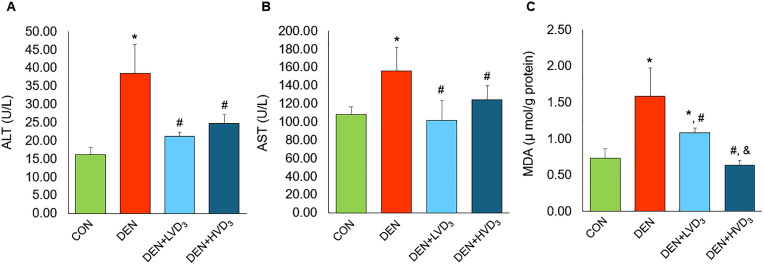
Effects of calcitriol on serum ALT, AST, and hepatic MDA levels in DEN-induced liver fibrosis. **(A)** ALT, **(B)** AST, and **(C)** hepatic MDA levels in the CON, DEN, DEN + LVD₃, and DEN + HVD₃ group after 8 weeks. DEN significantly increased ALT, AST, and MDA levels. Calcitriol attenuated ALT and AST similarly at both low and high doses, while the high dose produced a greater reduction in MDA than the low dose. Data are presented as mean ± SD, n = 6/group. ^*^p < 0.05 when compared with the CON group, ^#^*p* < 0.05 when compared with the DEN group, ^&^*p* < 0.05 when com*p*ared with the DEN + LVD_3_.

Serum ALT and AST levels were significantly increased in the DEN group compared with the CON group (ALT: 38.50 ± 7.87 U/L vs. 16.17 ± 1.83 U/L; and AST: 156.00 ± 26.08 U/L vs. 107.83 ± 8.61 U/L, respectively; *p* < 0.05). Both serum ALT and AST levels were significantly reduced in the DEN + LVD_3_ and DEN + HVD_3_ groups compared with the DEN group (ALT: 21.17 ± 1.17 U/L and 24.67 ± 2.42 U/L vs. 38.50 ± 7.87 U/L; and AST: 101.50 ± 21.96 U/L and 124.33 ± 15.65 U/L vs. 156.00 ± 26.08 U/L, respectively; *p* < 0.05) as shown in Fig 2A and 2B.

Hepatic MDA levels were significantly increased in the DEN and DEN + LVD_3_ groups compared with the CON group (1.59 ± 0.39 and 1.08 ± 0.07 vs. 0.73 ± 0.13 µmol/g protein, respectively; *p* < 0.05). Both DEN + LVD_3_ and DEN + HVD_3_ groups exhibited significantly lower MDA levels than the DEN group (1.08 ± 0.07 and 0.64 ± 0.07 vs. 1.59 ± 0.39 µmol/g protein, respectively; *p* < 0.05). Notably, the DEN + HVD_3_ group also exhibited significantly lower MDA levels than in the DEN + LVD_3_ group (0.64 ± 0.07 vs. 1.08 ± 0.07 µmol/g protein; *p* < 0.05), as shown in Fig 2C.

### Effects of calcitriol on liver morphology and histopathology in DEN-induced liver fibrosis

**Fig 3:**
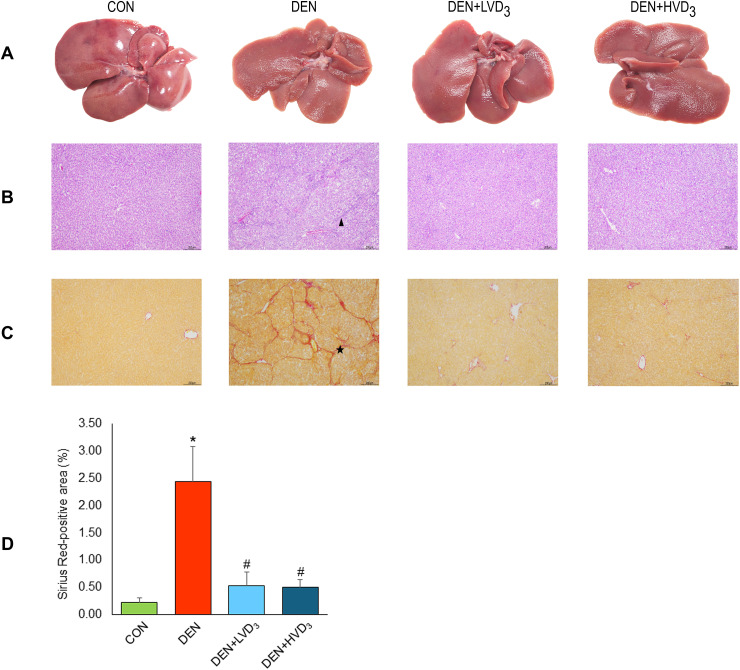
Effects of calcitriol on liver morphology, histological staining, and quantification of Sirius Red-positive area in DEN-induced liver fibrosis. **(A)** Representative images of gross liver morphology, **(B)** H&E staining, **(C)** Sirius Red staining for collagen deposition, **(D)** quantification of Sirius Red-positive area (%). Arrowhead indicates fibrotic regions; star indicates collagen deposition. Images were shown at 4x magnification. DEN induced a rough liver surface and increased collagen deposition, while calcitriol improved gross morphology and reduced fibrosis. Data are presented as mean ± SD, n = 6/group. *p < 0.05 when com*p*ared with the CON group, ^#^*p* < 0.05 when com*p*ared with the DEN group.

The gross appearance of the liver revealed a smooth surface in the control group, whereas a rough surface was observed in the DEN group. In contrast, calcitriol administration resulted in a noticeably smoother liver surface compared with the DEN group, as shown in Fig 3A.

Histopathological assessment using H&E and Sirius Red staining demonstrated normal liver architecture in the control group. In contrast, the DEN group exhibited increased fibrosis. Sirius Red staining showed prominent red collagen deposition in the DEN group. Both calcitriol-treated groups demonstrated a reduction in fibrosis, as shown in Figs 3B and 3C. Quantitatively, the Sirius Red-positive area (indicating collagen deposition) was significantly higher in the DEN group compared with the CON group (2.44 ± 0.64% vs. 0.23 ± 0.08%; *p* < 0.05). The Sirius Red-*p*ositive areas in the DEN + LVD_3_ and DEN + HVD_3_ groups showed a markedly decrease compared with the DEN group (0.53 ± 0.25% and 0.50 ± 0.14% vs. 2.44 ± 0.64%, respectively; *p* < 0.05), as shown in Fig 3D.

Liver fibrosis was evaluated using the METAVIR scoring system (Bedossa & Poynard, 1996) [44], which, although originally developed for human liver biopsies, has been adapted for rodent models to semi-quantitatively assess fibrosis based on collagen deposition and architectural distortion. Adapted METAVIR scores were assigned as follows; F0 = no fibrosis, F1 = fibrosis with portal zone expansion, F2 = fibrosis with expansion of most portal zones, and occasional bridging, F3 = fibrosis with expansion of most portal zones, marked bridging, and occasional nodules, F4 = cirrhosis [52].

In this study, all control rats were classified as F0. The DEN group showed the highest fibrosis severity, with four rats graded as F3, one as F2, and one as F1. In contrast, calcitriol-treated groups exhibited markedly lower fibrosis scores: in the DEN + LVD_3_ group, four rats were F0 and two were F1, while in the DEN + HVD_3_ group, five rats were F0, one was F1 (Table 1).

**Table 1:** Fibrosis scores across experimental groups.

Group	n	Fibrosis score (METAVIR system)					*p*-value
		F0	F1	F2	F3	F4	
CON	6	6	–	–	–	–	
DEN	6	–	1	1	4	–	0.007*
DEN + LVD_3_	6	4	2	–	–	–	0.025^#^
DEN + HVD_3_	6	5	1	–	–	–	0.019^#^

Data are presented as the number of rats in each fibrosis grade. Control rats were all F0. DEN induced marked fibrosis (F1-F3), whereas calcitriol treatment reduced fibrosis, with most rats in the DEN + LVD_3_ and DEN + HVD_3_ groups classified as F0-F1. Statistical analysis was performed using Pearson’s Chi-Square test, *p < 0.05 when compared with the CON group, ^#^*p* < 0.05 when compared with the DEN group.

### Effects of calcitriol on NF-κB p65 expression in DEN-induced liver fibrosis

**Fig 4:**
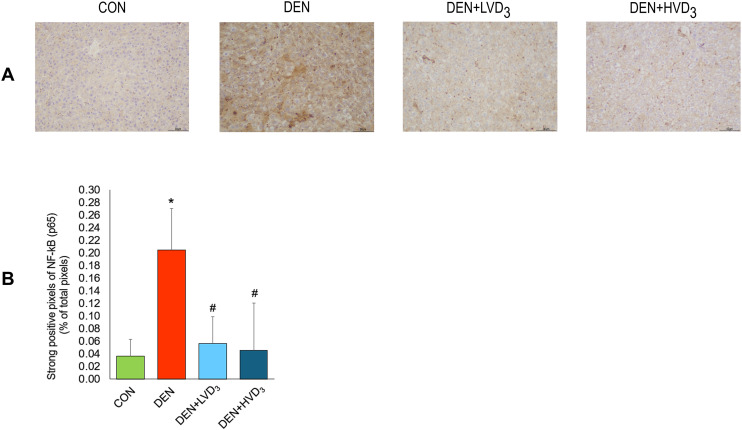
Effects of calcitriol on hepatic NF-κB p65 expression in DEN-induced liver fibrosis. **(A)** Representative immunohistochemistry images of NF-κB p65 expression. Brown staining indicates positive expression (images shown at 10x magnification). **(B)** Quantification of strongly positive NF-κB p65 staining (% of total pixels). DEN markedly increased NF-kB p65 expression compared with CON, while both low- and high-dose calcitriol reduced NF-kB p65. Data are presented as mean ± SD, n = 6/group. *p < 0.05 when compared with the CON group, ^#^*p* < 0.05 when compared with the DEN group.

Immunohistochemical analysis of NF-κB p65 expression showed normal staining patterns in the control group, whereas the DEN group displayed markedly increased strong positive staining (brown coloration). In contrast, both calcitriol-treated groups (DEN + LVD_3_ and DEN + HVD_3_) showed a reduction in NF-κB p65 expression compared with the DEN group, as shown in Fig 4A.

The percentage of strongly positive pixels (number of strongly positive pixels/totals pixels) was significantly higher in the DEN group compared with the CON group (0.200 ± 0.066% vs. 0.037 ± 0.027%, respectively; *p* < 0.05). Interestingly, this percentage was markedly reduced in both the DEN + LVD_3_ and DEN + HVD_3_ groups compared with the DEN group (0.056 ± 0.043% and 0.046 ± 0.075% vs. 0.200 ± 0.066%, respectively; *p* < 0.05), as shown in Fig 4B.

### Effects of calcitriol on MMP-12, TIMP-1, α-SMA, and TGF-β1 expression in DEN-induced liver fibrosis

**Fig 5:**
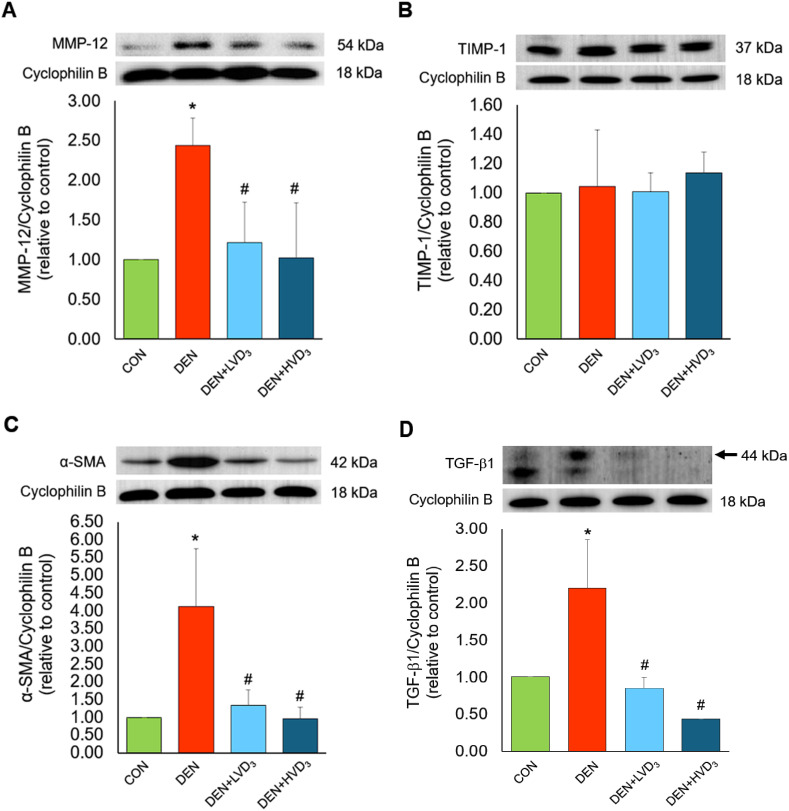
Effects of calcitriol on hepatic MMP-12, TIMP-1, α-SMA, and TGF-β1 protein expression in DEN-induced liver fibrosis. Representative western blot images and quantitative analyses for MMP-12 **(A)**, TIMP-1 **(B)**, α-SMA **(C)**, and TGF-β1 **(D)**. DEN significantly increased MMP-12, α-SMA, and TGF-β1 compared with CON, while calcitriol reduced their expression. TIMP-1 showed no significant changes across groups. Data are presented as mean ± SD, *p < 0.05 when compared with the CON group, ^#^*p* < 0.05 when compared with the DEN group.

The protein expression levels of MMP-12, α-SMA, and TGF-β1 (normalized to cyclophilin B) were significantly elevated in the DEN group compared with the control group (MMP-12/CPB; 2.44 ± 0.34 vs. 1.00 ± 0.00, and α-SMA/CPB; 4.13 ± 1.63 vs. 1.00 ± 0.00, and TGF-β1/CPB; 2.20 ± 0.65 vs. 1.00 ± 0.00, respectively; *p* < 0.05). Both calcitriol-treated groups (DEN + LVD_3_ and DEN + HVD_3_ groups) showed significantly reduced the expression of these fibrosis-related proteins compared with the DEN group (MMP-12/CPB; 1.21 ± 0.51 and 1.02 ± 0.69 vs. 2.44 ± 0.34 and α-SMA/CPB; 1.35 ± 0.44 and 0.96 ± 0.33 vs. 4.13 ± 1.63, and TGF-β1/CPB; 0.85 ± 0.15 and 0.43 ± 0.00 vs 2.20 ± 0.65, respectively; *p* < 0.05), as shown in Fig 5A, 5C and 5D. In contrast, TIMP-1 expression showed no significant differences among the CON, DEN, DEN + LVD_3_, and DEN + HVD_3_ groups (TIMP-1/CPB; 1.00 ± 0.00 vs. 1.04 ± 0.39 vs. 1.01 ± 0.13 and 1.14 ± 0.14, respectively; *p* > 0.05), as shown in Fig 5B. Full western blot images are provided in the Supporting information: S1 File.

### Effects of calcitriol on hepatic SOD-1, and GPX-1 gene expression in DEN-induced liver fibrosis

**Fig 6:**
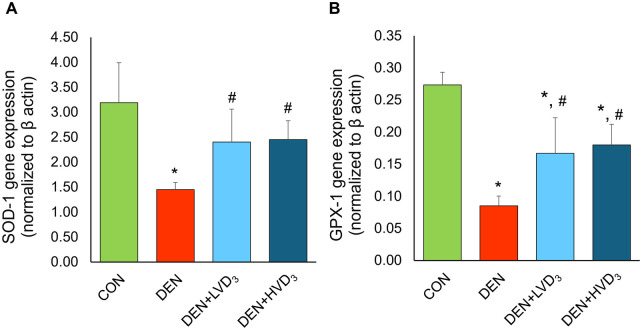
Effects of calcitriol on hepatic SOD-1, and GPX-1 gene expression in DEN-induced liver fibrosis. The levels of hepatic SOD-1 **(A)**, and GPX-1 **(B)** gene expression in all experimental groups. DEN significantly decreased SOD-1 and GPX-1 expression compared with CON. Calcitriol partially enhanced SOD-1 and GPX-1 levels. Data are presented as mean ± SD, n = 6/group. *p < 0.05 when compared with the CON group, ^#^*p* < 0.05 when compared with the DEN group.

Hepatic antioxidant gene expressions were shown in Fig 6A and 6B. SOD-1 expression (normalized to β-actin) was significantly reduced in the DEN group compared with the control group (1.45 ± 0.14 vs. 3.19 ± 0.80), *p* < 0.05). Calcitriol treatment (DEN + LVD3 and DEN + HVD3) significantly increased SOD-1 expression compared with the DEN group (2.40 ± 0.66 and 2.45 ± 0.38 vs 1.45 ± 0.14, respectively; *p* < 0.05), as shown in Fig 6A.

GPX-1 expressions were also significantly decreased in the DEN group compared with controls (0.09 ± 0.02 vs. 0.27 ± 0.02, *p* < 0.05). Both calcitriol doses (DEN + LVD_3_ and DEN + HVD_3_) significantly attenuated the DEN-induced reduction in GPX-1 expression compared with the DEN group (0.17 ± 0.06 and 0.18 ± 0.03 vs. 0.09 ± 0.02, respectively; *p* < 0.05), although levels remained lower than controls as shown in Fig 6B.

## Discussion

Vitamin D has been proposed as a potential therapeutic agent due to its antioxidant and metabolic effects, which may counteract oxidative stress, a key driver of DEN-induced liver fibrosis. In this study, DEN-treated rats exhibited significant body weight loss (469 g), approximately 20% lower than controls (582 g), reflecting DEN-associated metabolic stress and malnutrition. Weight loss is a common manifestation of liver fibrosis and may result from reduced appetite and discomfort associated with liver injury [53]. Consistent with previous reports, our study showed that DEN reduced body and liver weights in DEN-treated rats compared to controls [54]. Calcitriol-treated rats showed similar weight reductions (472 g for low-dose, 463 g for high-dose), corresponding to 18–21% below controls, with no significant differences compared to DEN alone. These findings suggest that weight loss was primarily due to DEN-induced toxicity, which cannot be counteracted by calcitriol administration. This finding was consistent with the results by Eitah et al., which showed no significant effects of oral vitamin D (200 IU/kg/day) on body or liver weights in DEN-treated rats [55]. The calcitriol doses used in this study (5 µg and 10 µg, approximately 200 and 400 IU) are considered physiologically safe. Nevertheless, vitamin D₃ has been reported to modulate lipid metabolism by downregulating sterol regulatory element-binding proteins (SREBPs) [56] and to improve insulin sensitivity, which may contribute to reduced appetite and food intake [57]. However, unlike our findings, a previous study reported an increased relative liver weight/body weight in mice with DEN-induced liver preneoplasia treated orally with vitamin D (200 IU/kg/day). Vitamin D was administered either 2 weeks before DEN injection (Vit D + DEN, first protocol) or 1 week after the first DEN dose (DEN + Vit D, second protocol). In both protocols, vitamin D caused a non-significant increase in absolute liver weight but normalized the relative liver weight [55], highlighting potential for dose- and duration-dependent effect.

Liver injury after 8 weeks of DEN administration was evaluated using serum ALT and AST levels. DEN is metabolized by CYP2E1, generating ROS that induce oxidative stress and DNA damage, contributing to hepatocellular necrosis, fibrosis, cirrhosis, and HCC [58]. Consistent with previous studies, DEN significantly increased ALT and AST levels [7,55], indicating hepatocyte membrane damage and enzymes leakage [59]. DEN also markedly increased hepatic MDA levels, indicating enhanced oxidative stress and impaired antioxidant defenses [60], consistent with observations in other fibrosis models, including high-fat diet induced NAFLD [19]. ROS generated during DEN metabolism further activate pro-inflammatory pathways, particularly NF-κB signaling [61]. Even a single low-dose DEN exposure (10 mg/kg for two weeks) significantly increases NF-κB expression, highlighting the interplay between oxidative stress and inflammation in DEN-induced liver injury [61]. In this study, calcitriol significantly reduced serum ALT, AST, hepatic MDA levels, and NF-κB p65 expression, indicating a protective effect against DEN-induced hepatotoxicity and attenuation of oxidative stress. Notably, the higher calcitriol dose (10 μg/kg BW) produced a greater reduction in hepatic MDA than the lower dose (5 μg/kg BW), contrasting with findings by Ma et al., who reported greater MDA reduction with the lower vitamin D_3_ dose (5 μg/kg) [19]. Vitamin D₃ may suppress NF-κB activity either directly through the calcitriol/vitamin D receptor (VDR) complex or indirectly via associated signaling pathways [62]. Activation of mitogen-activated protein kinase (MAPK), can also stimulate NF-kB and upregulated antioxidant enzymes [63], representing a protective mechanism activated in response to cellular stress to upregulate defend molecules. These suggests that antioxidant responses to vitamin D may vary depending on disease model, exposure duration, or the form of vitamin D administered.

The imbalance between ROS production and antioxidant defenses is a hallmark of DEN-induced hepatotoxicity. Superoxide dismutase (SOD) is the primary enzymatic defense against oxidative stress by catalyzing the conversion of superoxide anions (O_2_•-) into hydrogen peroxide (H_2_O_2_) and molecular oxygen (O_2_) [64]. Excessive superoxide generation accelerates SOD consumption and disrupts redox homeostasis. In our study, DEN exposure significantly reduced hepatic SOD-1 gene expression, indicating compromised antioxidant defenses, likely due to increased SOD utilization during DEN metabolism [65]. Previous study has shown that hepatocyte specific SOD1-deficiency or combined SOD1/2 deficiencies exacerbate oxidative injury and steatohepatitis in high-fat-fed mice [66], highlighting the critical role of superoxide detoxification. Similarly, DEN suppressed glutathione peroxidase-1 (GPX-1), a key enzyme responsible for detoxifying H_2_O_2_ and maintaining redox homeostasis [67,68], and moderately affected glutathione S-transferases A1 (GSTA-1), a phase II detoxification enzyme involved in conjugating reduced glutathione (GSH) to reactive intermediates and protects against oxidative stress [69]. The present study demonstrates that calcitriol reduced hepatic MDA levels and enhanced SOD-1 and GPX-1 gene expression, indicating a partial re-establishment of hepatic antioxidant capacity. Vitamin D deficiency has previously been shown to downregulate antioxidant enzymes, including SOD and GPX [70], supporting the responsiveness of these pathways to vitamin D status. In our study, calcitriol partially enhanced GPX-1 expression, indicating reinforcement of the hepatic antioxidant defense system. These findings are consistent with evidence that vitamin D enhanced GPX activity and glutathione metabolism, through activation of the Nrf2 pathway [71], which regulates the transcription of multiple antioxidant enzymes [72]. Although GPX-1 expression in the calcitriol-treated groups did not fully return to control levels, this may reflect a regulated adaptive response. Previous study has reported that GPX-1 deficiency can paradoxically reduce fibrosis by modulating fibrogenic signaling pathways (TGF-β1, α-SMA) [73], highlighting the context-dependent role of GPX-1 in liver pathology. Thus, the partial enhancement of GPX-1 observed here may represent an adaptive protective response against fibrogenesis. These findings suggest that calcitriol mitigates DEN-induced oxidative stress by upregulating key antioxidant genes (SOD-1, GPX-1), thereby reducing hepatocellular damage and providing mechanistic support for its antifibrotic effects.

Fibrogenesis in DEN-induced liver injury is primarily driven by HSC activation, as demonstrated by the marked increases in α-SMA expression and collagen deposition. Activated HSCs produce excessive ECM, and profibrotic mediators such as TGF-β1 further amplify this response [33]. The elevated α-SMA observed in this study reflects active fibrogenesis, and the increase in MMP-12 indicates an attempt to degrade accumulated ECM during injury. However, the concurrent upregulation of MMP-12 and its inhibitor TIMP-1 suggests dysregulated matrix remodeling, consistent with impaired ECM turnover characteristic of chronic DEN-induced fibrosis [34,35]. Calcitriol ameliorated these fibrotic changes. Its antifibrotic effects are likely mediated through activation of the VDR, which is expressed in hepatocytes, Kupffer cells, and most prominently in HSCs. VDR signaling suppresses HSC activation by antagonizing TGF- β/Smad pathways and limiting ECM gene transcription [74]. Consistent with these mechanisms, calcitriol reduced α-SMA and TGF-β1 expressions and decreased collagen deposition in DEN-treated rats. Calcitriol also lowered MMP-12 levels without altering TIMP-1, suggesting partial normalization of ECM remodeling dynamics.

Together, these data indicate that calcitriol mitigated DEN-induced fibrogenesis by reducing oxidative stress, suppressing HSC activation, and modulation ECM turnover. Because both doses produced comparable antifibrotic effects, differing primarily in their impact on MDA reduction, the 5 µg/kg BW dose appears sufficient to achieve therapeutic benefit while minimizing unnecessary high dosing and cost. Although serum calcium and phosphorus were not directly assessed in the present study, Goto et al. (2022) demonstrated that substantially higher doses of vitamin D₃ did not induce hypercalcemia or hyperphosphatemia in DEN/TAA-treated animals [7]. Therefore, the much lower calcitriol doses used here are unlikely to produce vitamin D–related toxicity. Furthermore, inflammation was evaluated using a single marker, which may not fully capture the complexity of inflammatory signaling. Inclusion of broader inflammatory markers in future studies would strengthen mechanistic interpretation.

## Conclusions

DEN administration induced pronounced oxidative stress, lipid peroxidation (MDA), and hepatocellular injury (ALT, AST), leading to activation of inflammatory (NF-κB p65) and fibrogenic pathways (TGF-β1, MMP-12) and subsequent hepatic stellate cell activation, myofibroblast transition, and ECM deposition (α-SMA, collagen). Calcitriol attenuated these DEN-induced effects by reducing oxidative stress and lipid peroxidation while upregulating key antioxidant defenses (SOD-1, GPX-1), thereby suppressing downstream inflammatory and fibrogenic signaling and limiting fibrosis progression (TGF-β1, MMP-12, α-SMA, collagen). Collectively, these findings support a protective role for calcitriol against toxin-induced liver fibrosis through coordinated antioxidant and antifibrotic mechanisms, highlighting its potential relevance for attenuating fibrosis progression during early-stage disease.

## Supporting information

S1 Fig Sample size calculation using G*Power software (version 3.1.9.4). As the MDA levels was one of our primary outcomes, data from Megahed et.al., (2023) were used as the reference, with α = 0.05 and power = 0.95., yielding six animals per group (total n = 24).
(TIF)

S2 Fig Weekly body weights for all experimental groups. The graph shows weekly body weights for all groups over the 8-week period. DEN-treated rats exhibited lower body weights than controls from week 3 onward, with this difference was maintained throughout the study. However, no significant differences were observed among DEN groups with or without calcitriol administration. Data are expressed as mean ± SD (n = 6/group). *p < 0.05 compared with the control group.
(TIF)

S1 Table Specific primer sequences used for quantitative real-time PCR (qRT-PCR) analysis of target genes. Gene names and corresponding gene IDs for SOD-1, GPX-1, and β-actin (internal control) are provided. Detailed primer information was streamlined in the Supporting Information for clarity.
(PDF)

S1 File Original uncropped western blot images showing detection bands for MMP-12, TIMP-1, α-SMA, TGF-β1, and CPB. For the MMP-12/CPB blot only, lane 1 contains the PM2600 ExcelBand™ 3-Color High Range protein marker (SMOBIO, Taiwan), lanes 2–5 correspond to the control, DEN, DEN + low-dose calcitriol (5 μg/kg body weight), and DEN + high-dose calcitriol (10 μg/kg body weight) groups, respectively, and lane 6 contains the protein marker. For all other blots, lanes correspond to the experimental groups as indicated in the figure. Blots were developed using Clarity Western ECL substrates (BioRad, California, USA), captured with the Bio-Rad ChemiDoc Touch Imaging System, and quantified using Image Lab software (Bio-Rad, California, USA). Processed images were exported as TIF files for publication.
(PDF)

## Abbreviations

α-SMA: alpha smooth muscle actin
DEN: diethylnitrosamine
GPX: Glutathione peroxidase
MDA: malondialdehyde
NF-κB p65: nuclear factor kappa B p65
MMP-12: matrix metalloproteinase 12
SOD: Superoxide dismutase
TIMP-1: tissue inhibitor of metalloproteinase 1
TGF-β: Transforming growth factor-beta
