# Supplementary figures and images for: Calcitriol attenuates diethylnitrosamine-induced hepatic fibrosis in rats by reducing oxidative stress and fibrogenic mediators
Source: PLoS One. 2026 May 6;21(5):e0347908. doi: 10.1371/journal.pone.0347908 (PMC13148716; doi:10.1371/journal.pone.0347908)

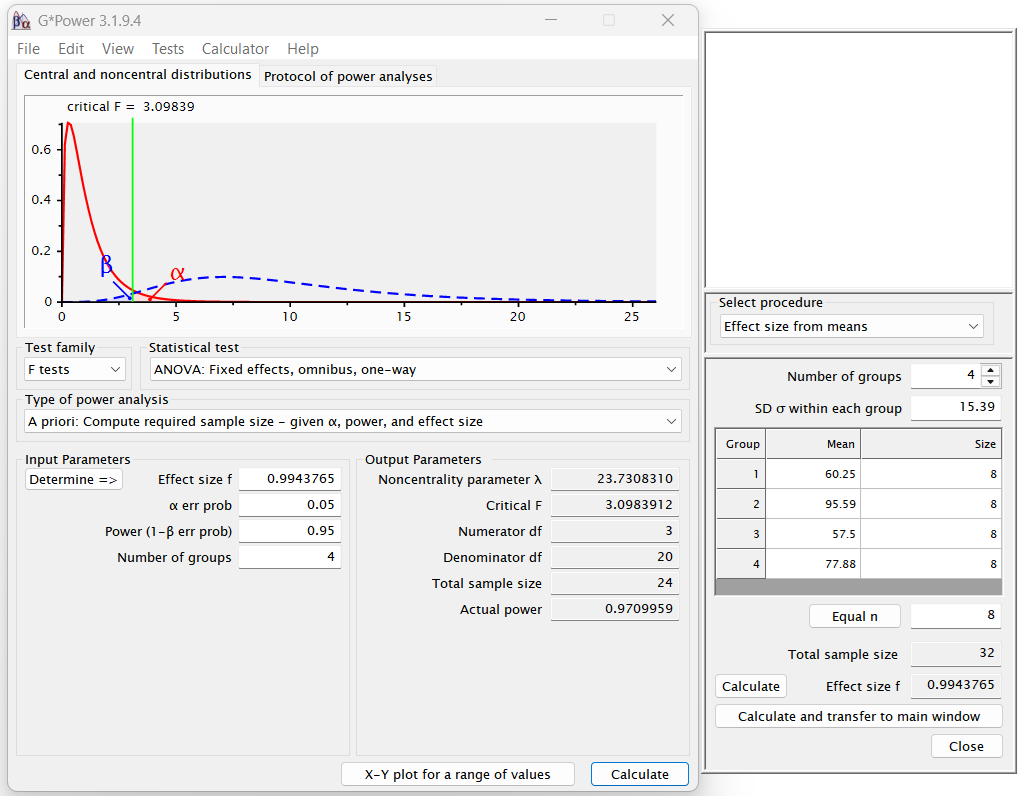

Supplement: S1 Fig — As the MDA levels was one of our primary outcomes, data from Megahed et.al., (2023) were used as the reference, with α = 0.05 and power = 0.95., yielding six animals per group (total n = 24). (TIF) [file pone.0347908.s001.tif]

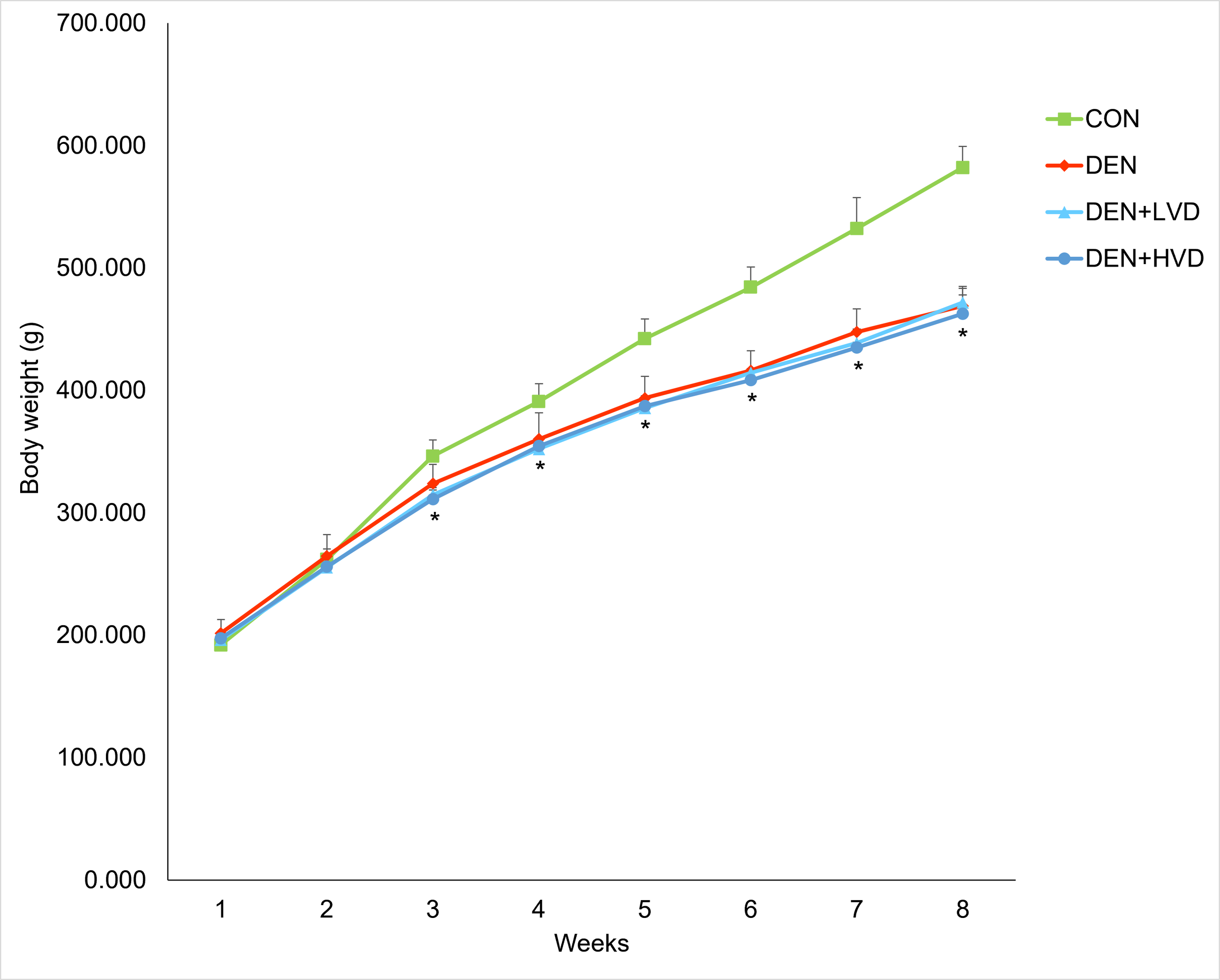

Supplement: S2 Fig — The graph shows weekly body weights for all groups over the 8-week period. DEN-treated rats exhibited lower body weights than controls from week 3 onward, with this difference was maintained throughout the study. However, no significant differences were observed among DEN groups with or without calcitriol administration. Data are expressed as mean ± SD (n = 6/group). *p < 0.05 compared with the control group. (TIF) [file pone.0347908.s002.tif]
