# Supplementary material for: Calcitriol attenuates diethylnitrosamine-induced hepatic fibrosis in rats by reducing oxidative stress and fibrogenic mediators
Source: PLoS One. 2026 May 6;21(5):e0347908. doi: 10.1371/journal.pone.0347908 (PMC13148716; doi:10.1371/journal.pone.0347908)
Supplement: S1 Table — Gene names and corresponding gene IDs for SOD-1, GPX-1, and β-actin (internal control) are provided. Detailed primer information was streamlined in the Supporting Information for clarity. (PDF) [file pone.0347908.s003.pdf]

S3 Table. Specific primer sequences for the expression analysis of the target genes by qRT-PCR.

Gene and Gene ID	NCBI Reference Sequence	Primer sequences
SOD-1- 24786	NM_017050.1	F: 5'-TATGGTGGTCCACGAGAAAC-3'<br>R: 5'-AATCACACCACAAGCCAAGC-3'
GPX-1- 24404	NM_030826.4	F: 5'-TCAGTTCGGACATCAGGAGAATGG-3'<br>R: 5'-GGATCGTCACTGGGTGCTGG-3'
β-Actin- 728378	NM_001099771.1	F: 5'-ACGGTCAGGTCATCACTATCG-3'<br>R: 5'-GGCATAGAGGTCTTTACGGATG-3'
