# Supplementary material for: Calcitriol attenuates diethylnitrosamine-induced hepatic fibrosis in rats by reducing oxidative stress and fibrogenic mediators
Source: PLoS One. 2026 May 6;21(5):e0347908. doi: 10.1371/journal.pone.0347908 (PMC13148716; doi:10.1371/journal.pone.0347908)

Original uncropped blot images showing detection bands for MMP-12 and CPB.

A

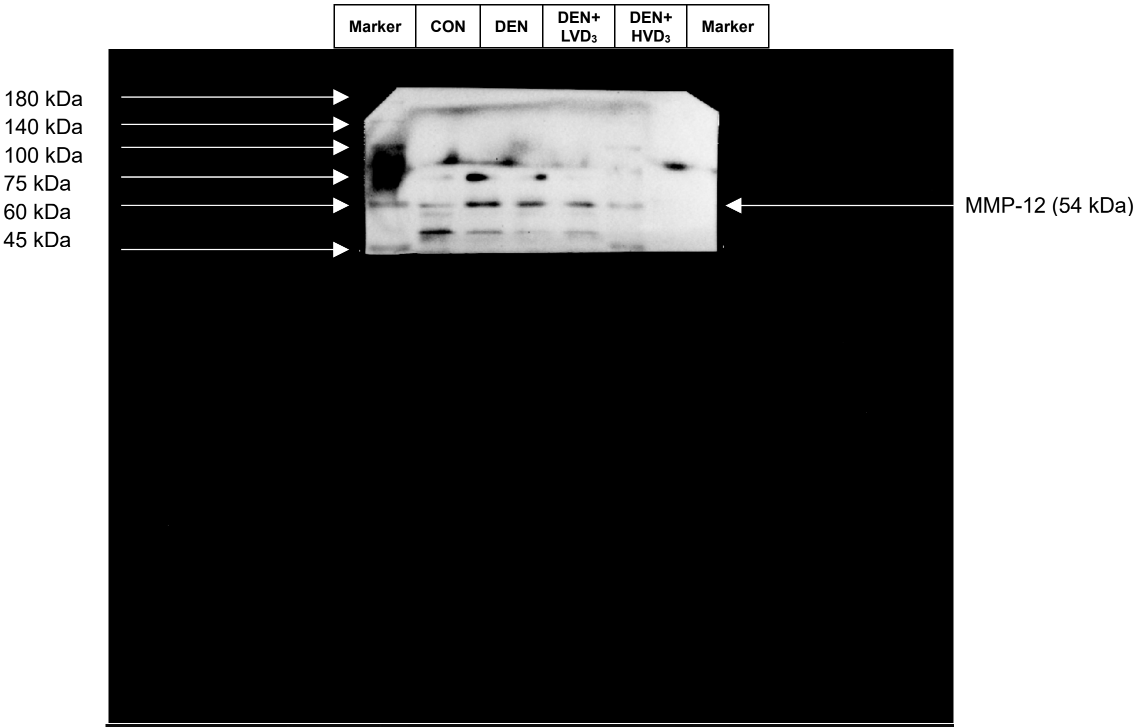

B

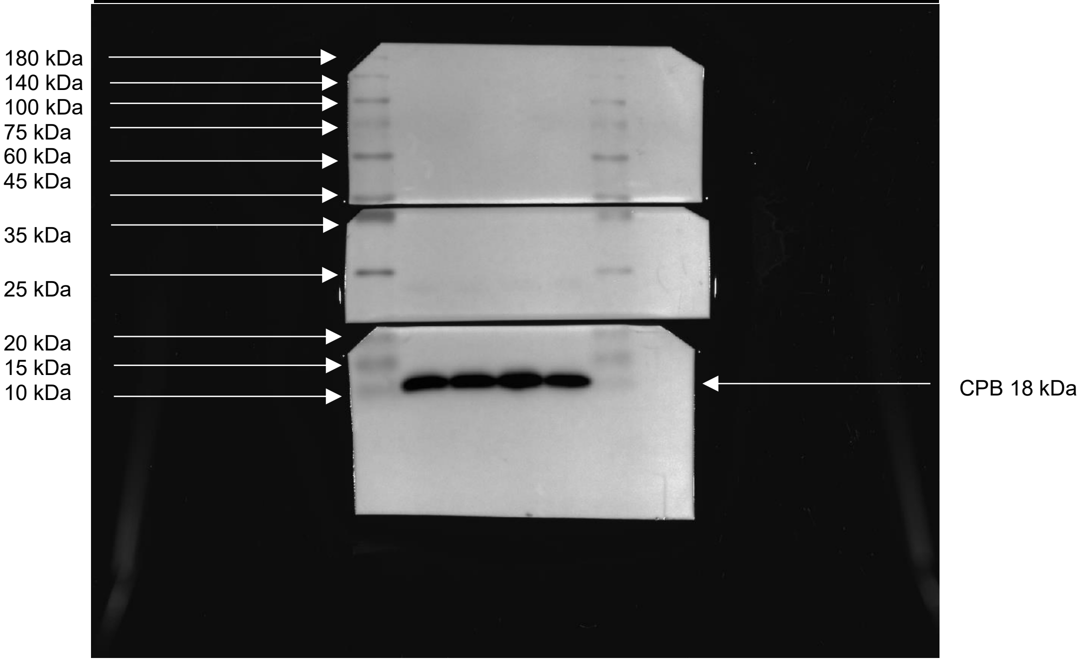

Original uncropped blot images showing detection bands for TIMP-1 and CPB.

A

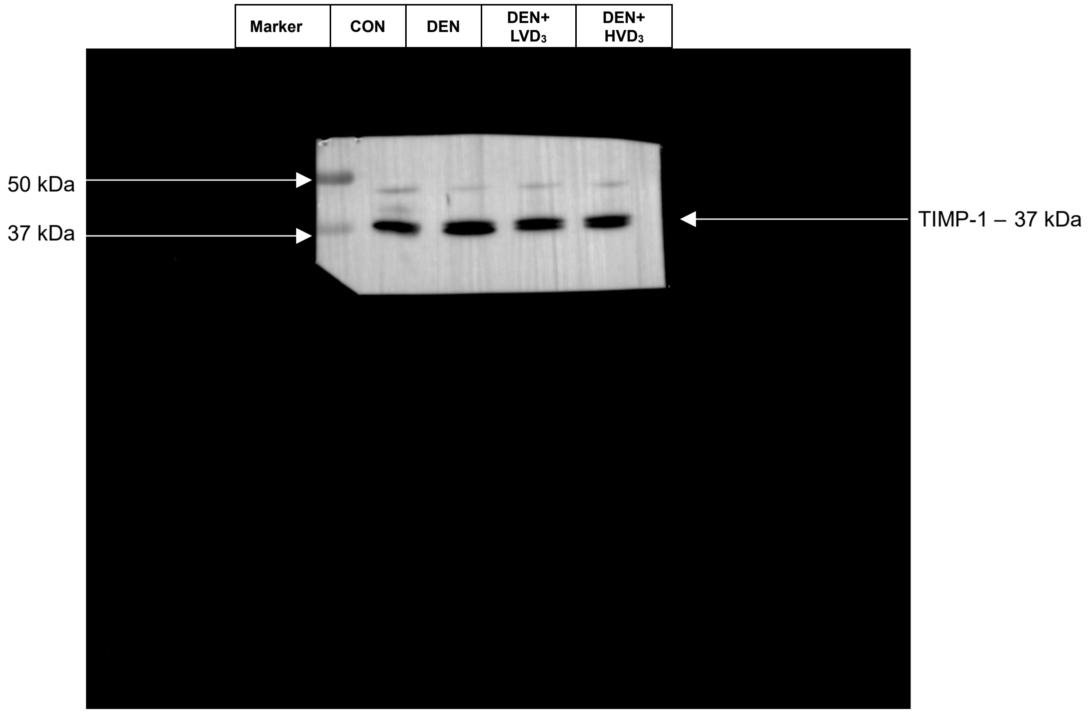

B

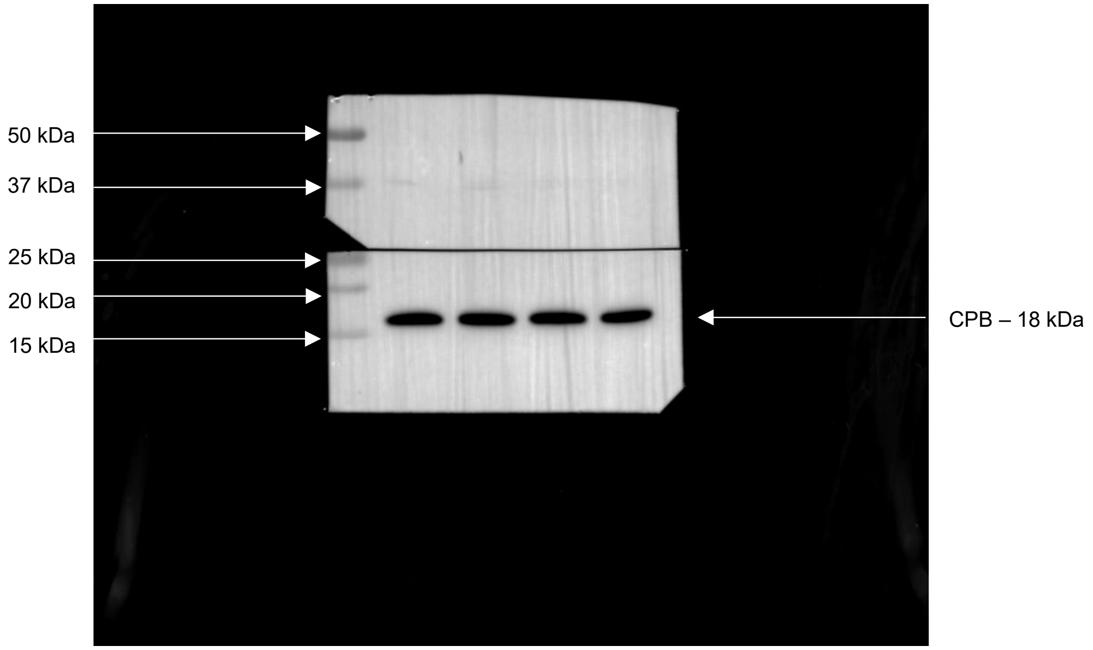

Original uncropped blot images showing detection bands for α-SMA and CPB.

A

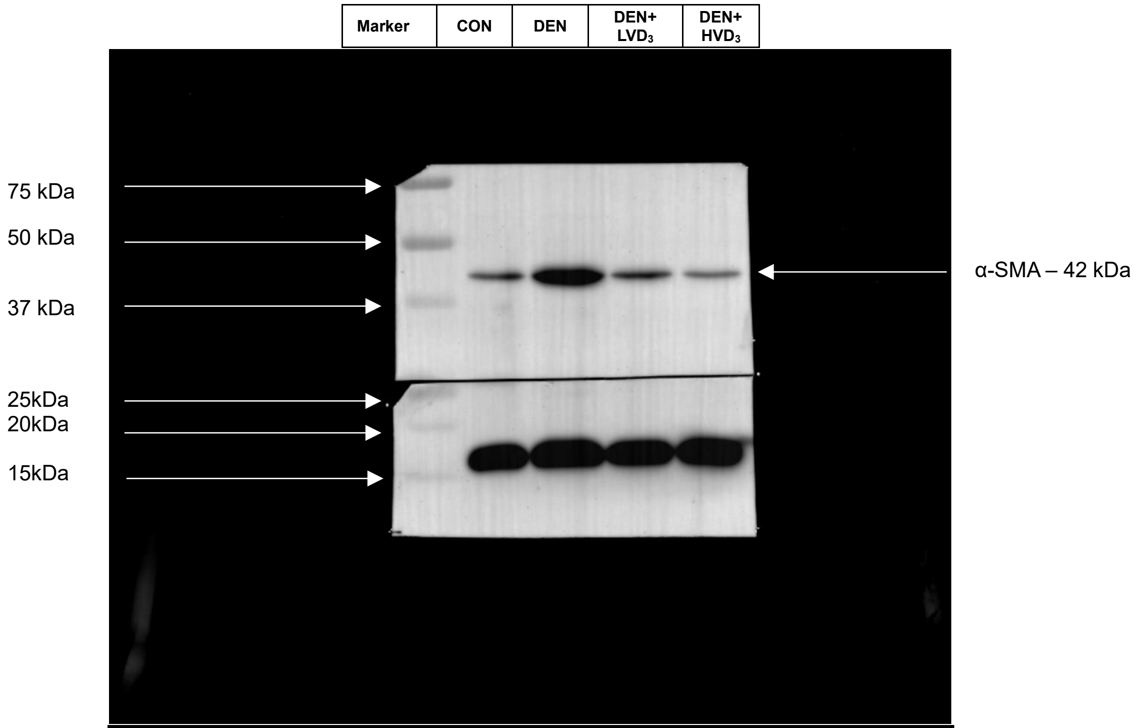

B

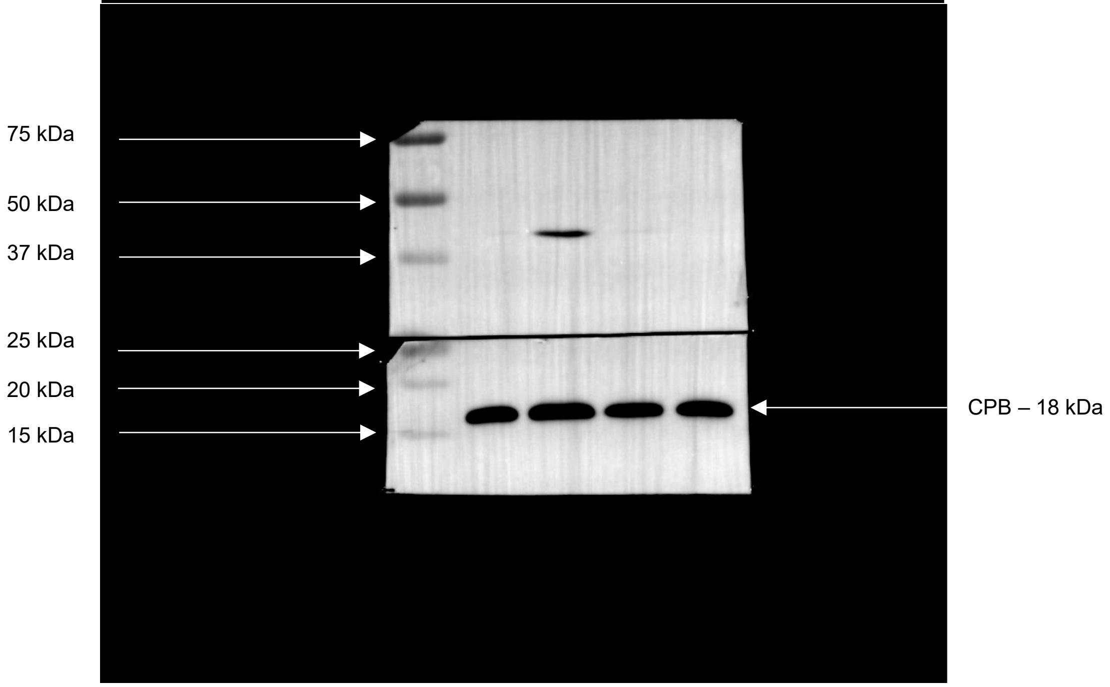

Original uncropped blot images showing detection bands for TGF-β1 and CPB.

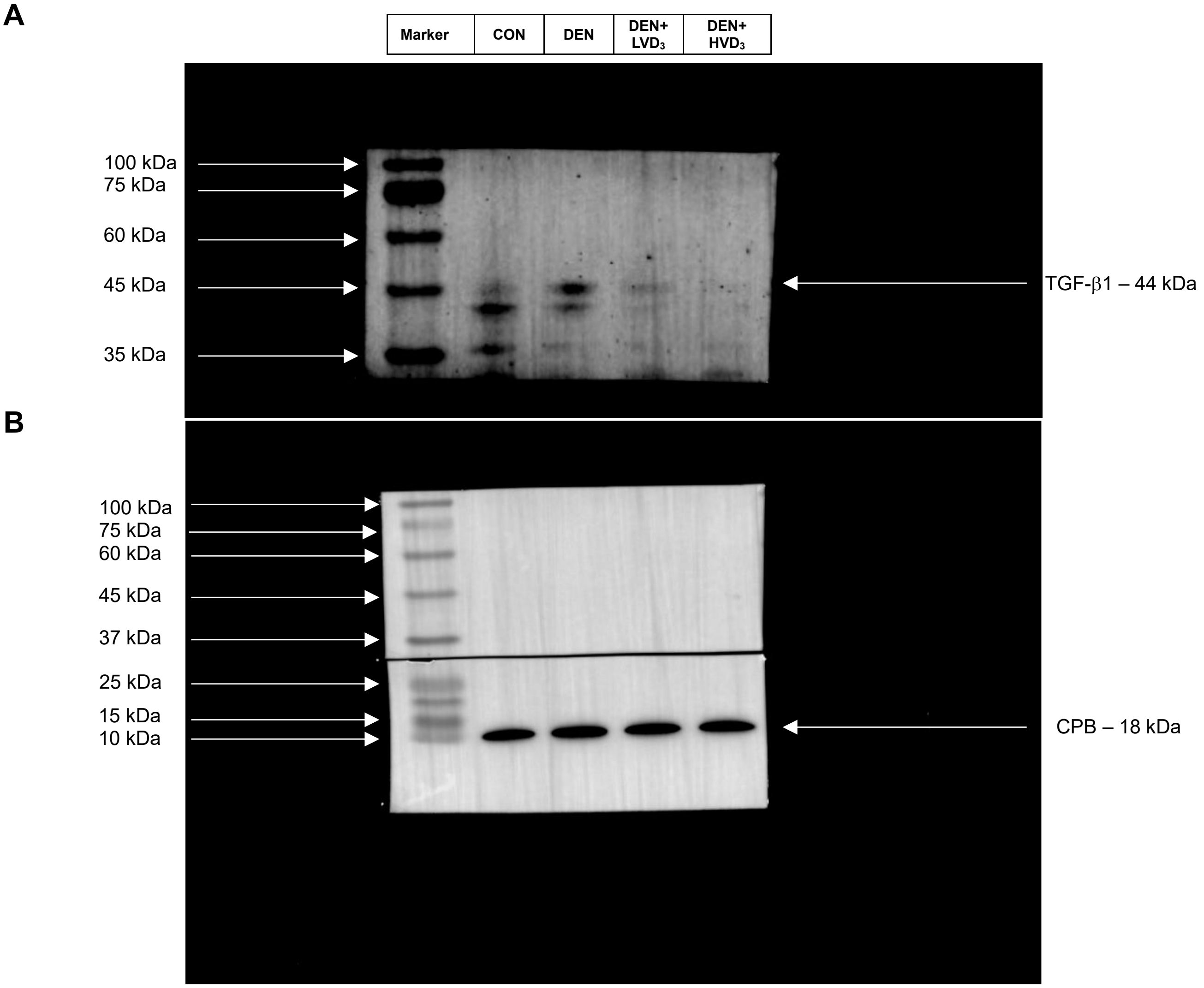

Supplement: S1 File — For the MMP-12/CPB blot only, lane 1 contains the PM2600 ExcelBand™ 3-Color High Range protein marker (SMOBIO, Taiwan), lanes 2–5 correspond to the control, DEN, DEN + low-dose calcitriol (5 μg/kg body weight), and DEN + high-dose calcitriol (10 μg/kg body weight) groups, respectively, and lane 6 contains the protein marker. For all other blots, lanes correspond to the experimental groups as indicated in the figure. Blots were developed using Clarity Western ECL substrates (BioRad, California, USA), captured with the Bio-Rad ChemiDoc Touch Imaging System, and quantified using Image Lab software (Bio-Rad, California, USA). Processed images were exported as TIF files for publication. (PDF) [file pone.0347908.s004.pdf]
